# Automatic human identification based on dental X-ray radiographs using computer vision

**DOI:** 10.1038/s41598-020-60817-6

**Published:** 2020-03-02

**Authors:** Andreas Heinrich, Felix V. Güttler, Sebastian Schenkl, Rebecca Wagner, Ulf K.-M. Teichgräber

**Affiliations:** 1 Department of Radiology, Jena University Hospital – Friedrich Schiller University, Am Klinikum 1, 07747 Jena, Germany; 2 Institute of Forensic Medicine, Jena University Hospital – Friedrich Schiller University, Am Klinikum 1, 07747 Jena, Germany

**Keywords:** Translational research, Mathematics and computing

## Abstract

A person may be identified by comparison between ante- and post-mortem dental panoramic radiographs (DPR). However, it is difficult to find reference material if the person is unknown. This is often the case when victims of crime or mass disaster are found. Computer vision can be a helpful solution to automate the finding of reference material in a large database of images. The purpose of the present study was to improve the automated identification of unknown individuals by comparison of ante- and post-mortem DPR using computer vision. The study includes 61,545 DPRs from 33,206 patients, acquired between October 2006 and June 2018. The matching process is based on the Speeded Up Robust Features (SURF) algorithm to find unique corresponding points between two DPRs (unknown person and database entry). The number of matching points found is an indicator for identification. All 43 individuals (100%) were successfully identified by comparison with the content of the feature database. The experimental setup was designed to identify unknown persons based on their DPR using an automatic algorithm system. The proposed tool is able to filter large databases with many entries of potentially matching partners. This identification method is suitable even if dental characteristics were removed or added in the past.

## Introduction

In forensic odontology, the comparison between an ante-mortem and a post-mortem DPR is a reliable method for person identification, because each person’s dentition and dentures are unique, and because these structures are quite resistant to post-mortal changes like decomposition^1–6^ and influences like high temperature^7,8^. However, it is difficult to find the matching reference DPR if there is no limiting indication of identity. This is often the case when victims of crime or mass disaster are found. Subsequently, a time-consuming public search using images of the dental implant or post-mortem DPR is carried out with the aim to find, e.g., a dentist or dental technician who can provide relevant information.

The recent literature^9–14^ reports different approaches and algorithms for identifying individuals from DPRs. Most of the methods currently described extract the tooth contours^9,11–13^ and/or dental works^10,13^, i.e. fillings, from the DPR. For the matching process, the Hausdorff distance^9^, the Levenshtein distance^10^ or the Euclidean distance^12,13^ have been calculated for the features to get a parameter for the similarity of two DPRs. A more complex matching procedure was published by Barboza *et al*.^11^. For the contour comparison she used two different methods, the so-called Shape Context^15^ and the Beam Angle Statistics^16^. Oktay^14^ identified the position of each tooth using machine learning and graphical models. Furthermore, a descriptor of the Pyramidal Histogram of Oriented Gradients (PHOG)^17^ describes each tooth. For the matching process, the tooth appearance (similarity of the descriptors of two data sets) and the distance to the middle of mouth gap were compared. In all methods, high identification success rates of 83% (Zhou *et al*.^9^), 86% (Hofer *et al*.^10^), 55% (Barboza *et al*.^11^), 67% (Frejlichowski *et al*.^12^), 72% (Karunya *et al*.^13^) and 81% (Oktay^14^) were achieved; however, for the evaluation the number of DPRs and persons was very low (maximum 206 DPRs of 170 persons). A further restriction is that optimal conditions are required for the application of the methods currently described, i.e. very good contrast, a lot of teeth, no dental implants, and no deciduous teeth. Some methods^11,12^ also require manual input, which is not possible for a large number of DPRs. The potential of an automatic person identification tool using DPR has not yet been sufficiently studied.

Computer vision^18^ can be a helpful solution to automate the finding of a reference DPR in a large database of images. It concerns the automatic extraction, analysis and understanding of useful information from a single image or a sequence of images^19^. Computer vision is already used to identify persons, e.g. for face recognition and biometrics^20–23^. An application of computer vision methods of dental age estimation, based on the lower third right molar in DPR, was developed by Čular *et al*.^24^; with a database comprising 203 DPRs.

The purpose of this study was to improve the automated identification of unknown individuals by comparison of ante-mortem and post-mortem DPRs using computer vision. In this publication, the improvement of a preliminary study^25^ will be presented. The present study is the first in which post-mortem DPRs of three bodies were made. For this purpose, a new type of holding device was designed.

## Results

In this study, 43 of 43 (100%) individuals were successfully identified by comparison with the content of the feature database (61,545 DPRs of 33,206 persons). The persons were between 6 and 83 years old. The dental statuses of the 40 persons were very different, e.g. deciduous teeth, healthy 32 teeth, braces, fillings, dental bridges, tooth loss, root canal treatments, pivot teeth with and without crowns, as well as only a few teeth without dental work.

### Evaluation of 40 randomly selected test persons

**Figure 1:**
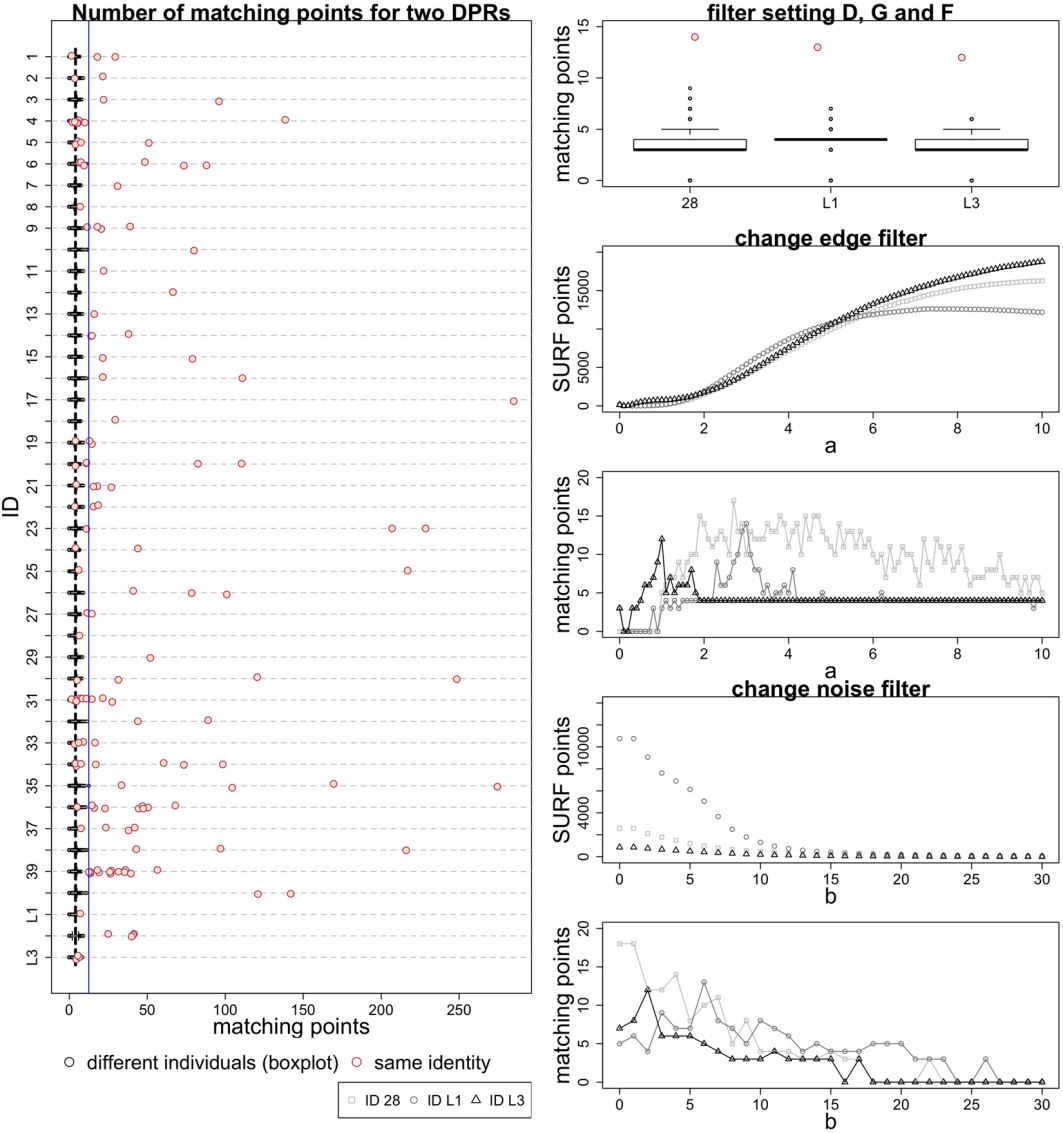
For the filter setting E (compare with Table 1), the maximum number of matching points for two DPRs of the same person (red points) and different individuals (black box plots) for all 40 randomly selected persons and three corpses (L1–3). A person has a red point for each of his/her own reference DPR. The threshold for the minimum number of matching points for unique identification is shown in blue. The identification process for ID 28, L1 and L3 did not succeed initially. For this reason, other filter settings were used to get an unambiguous result. The number of matching points found for the same person can depend directly on these filter settings.

In Fig. 1 (left), the average number of matching points found for the 40 persons by the method developed (filter setting E, see Table 1) was 52.01 ± 29.06 (maximum 285.00) for images of the same person. For non-identical individuals, the number was significantly and reliably lower, with 3.97 ± 0.63 matching points (maximum 12.50). Comparable results could be observed with other filter settings (see Table 2). Thereby, the method with filter setting E (see Table 1) yielded unambiguous identification for 38 out of 40 (95%) individuals with a minimum necessary number of 12.50 matching points (see Fig. 1, blue line) and 39 out of 40 (98%) individuals without consideration of a minimum number of matching points (see Table 3). The identification process for ID 28 did not succeed with filter setting E. For this reason, another filter was used (filter setting D or E2) to get an unambiguous result. The number of matching points found, and thus, unambiguous identification depends directly on the filter settings. All persons were identified with the filter setting E2; here, the main orientation of the features is ignored and the descriptor depends on the rotation. This approach seems to be an advantage for DPR, although the maximum of matching points for different identities is slightly higher (21 instead of 12.5). All 40 test persons (100%) were successfully identified by comparison with the content of the feature database (see Table 3).

**Table 1:** Main filter setting for eight systematic measurement series.

Vari-able	Description	Series of measurements with filter value						
		A	B	C	D	E/E2	F	G
*a*	Gradient of Sobel filter, larger value can detect more features	0	1	2	2	1.6	1	2.9
*b*	Size of averaging filter, larger value can reduce noise and signal processing time	0	0	4	4	3	2	6
*lev*	Number of scale levels (different box filters) per octave, larger value can detect more features at finer scale increments	4	4	4	8	11	6	4
*oct*	Number of octaves, larger value can detect larger features	3	3	3	6	14	2	5
*thr*	Metric threshold (minimum strength of the feature), larger value can reduce the number of features	1000	1000	1000	1500	600	1300	1000
*rot*	If true, the main orientation of the features is ignored and the descriptor is dependent on the rotation	false	false	false	true	false/true	false	false
*dis*	Maximum distance allowed between a point and the projection of its corresponding point (RANSAC)	10	10	10	15	10	12	8
*mat*	Order of matching functions (A = unknown person, B = database entry) with 1 = AB, 2 = BA, 3 = (AB+BA)/2, 4 = max(AB,BA), 5 = min(AB,BA)	3	3	3	1	3	5	2

The measurement series B corresponds to the filter setting from a preparatory study^25^. With a value of 0, the filter described was not applied.

**Table 2:** Result of the systematic measurement series (compare with Table 1) with MV = mean value, MED = median, MAX = maximum matching points, MAX (MV) = mean of maximum matching points per person for the same (=) identity and different (≠) identities.

Filter	Matching points							
	= identity				≠ identity			
	MV	MED	MAX	MAX (MV)	MV	MED	MAX	MAX (MV)
*A*	17.02 ± 6.03	16.36 ± 19.69	96.5	23.51 ± 23.34	2.76 ± 0.68	2.98 ± 0.16	8.5 (7)	5.28 ± 2.01
*B*	29.23 ± 16.09	28.04 ± 32.63	195	46.55 ± 48.30	3.54 ± 0.77	3.73 ± 0.34	11 (10)	7.40 ± 1.40
*C*	34.00 ± 18.13	32.45 ± 37.70	192	53.10 ± 45.90	3.64 ± 0.64	3.81 ± 0.27	10.5 (12)	7.39 ± 1.15
*D*	57.29 ± 35.62	54.90 ± 63.25	380	94.40 ± 91.57	4.15 ± 0.85	4.03 ± 0.36	21 (17)	12.15 ± 2.70
*E*	52.01 ± 29.06	50.08 ± 58.18	285	83.53 ± 77.98	3.97 ± 0.63	4.00 ± 0.00	12.5 (12.5)	8.75 ± 1.67
E2	68.77 ± 37.55	66.03 ± 75.51	399	110.40 ± 100.90	4.35 ± 1.03	4.35 ± 1.03	21 (21)	13.74 ± 3.21
*F*	30.14 ± 12.75	26.48 ± 32.94	201	45.92 ± 49.30	3.40 ± 0.47	3.45 ± 0.50	11 (10)	7.05 ± 1.77
*G*	33.08 ± 18.15	31.75 ± 36.15	182	52.10 ± 45.86	3.80 ± 0.48	4.00 ± 0.00	15 (13)	7.88 ± 1.64

The threshold for unambiguous identification is shown in column MAX for ≠ identity. The MAX result for 10 persons without reference DPR in the feature database is given in brackets.

**Table 3:** Summary of the identification results, signal processing time and database size of different measurement series (compare with Table 1).

*Filter*	*Successful identification Identities (images)*	>≠ identity (identified)	*ID not clearly identified (number of possible other identities)*	Signal processing time p. P. [min]	Database [GiB]
	>Threshold (unambiguous identification)				
*A*	27/40 (53/138) 0/3 corpse	34/40 (56/138) 1/3 corpse	2 (471), 7 (1), 8 (27476), 18 (65), 22 (1), 27 (13), L1 (4904), L3 (546)	8.56 ± 4.10	3.5
*B*	28/40 (68/138) 1/3	36/40 (78/138) 2/3	8 (12923), 13 (15), 19 (1), 31 (4), L1 (27243)	62.57 ± 25.45	12.4
*C*	35/40 (75/138) 1/3	39/40 (95/138) 1/3	8 (2062), L1 (11794), L3 (2606)	97.73 ± 21.60	17.5
*D*	33/40 (75/138) 1/3	39/40 (99/138) 2/3	8 (6590), L1 (13)	95.82 ± 25.51	24.8
*E*	38/40 (91/138) 1/3	39/40 (99/138) 1/3	28 (4), L1 (3), L3 (21)	298.94 ± 57.59	50.3
E2	36/40 (88/138) 1/3	40/40 (104/138) 3/3	all clearly identified	264.63 ± 60.41	49.1
F	29/40 (62/138) 2/3	33/40 (68/138) 2/3	2 (19495), 7 (13050), 8 (fail), 13 (23075), 19 (278), 27 (5), 28 (54), L1 (25254)	62.11 ± 29.09	10.9
G	31/40 (55/138) 1/3	38/40 (92/138) 2/3	8 (18030), 13 (6), L3 (25676)	161.45 ± 27.72	33.6
best	38/40 2/3	40/40 3/3			

In case of more than one positive result (not clearly identified), the number of potentially identical persons with the same or greater number of matching points is given in brackets. The threshold for unambiguous identification is shown in Table 2.

**Figure 2:**
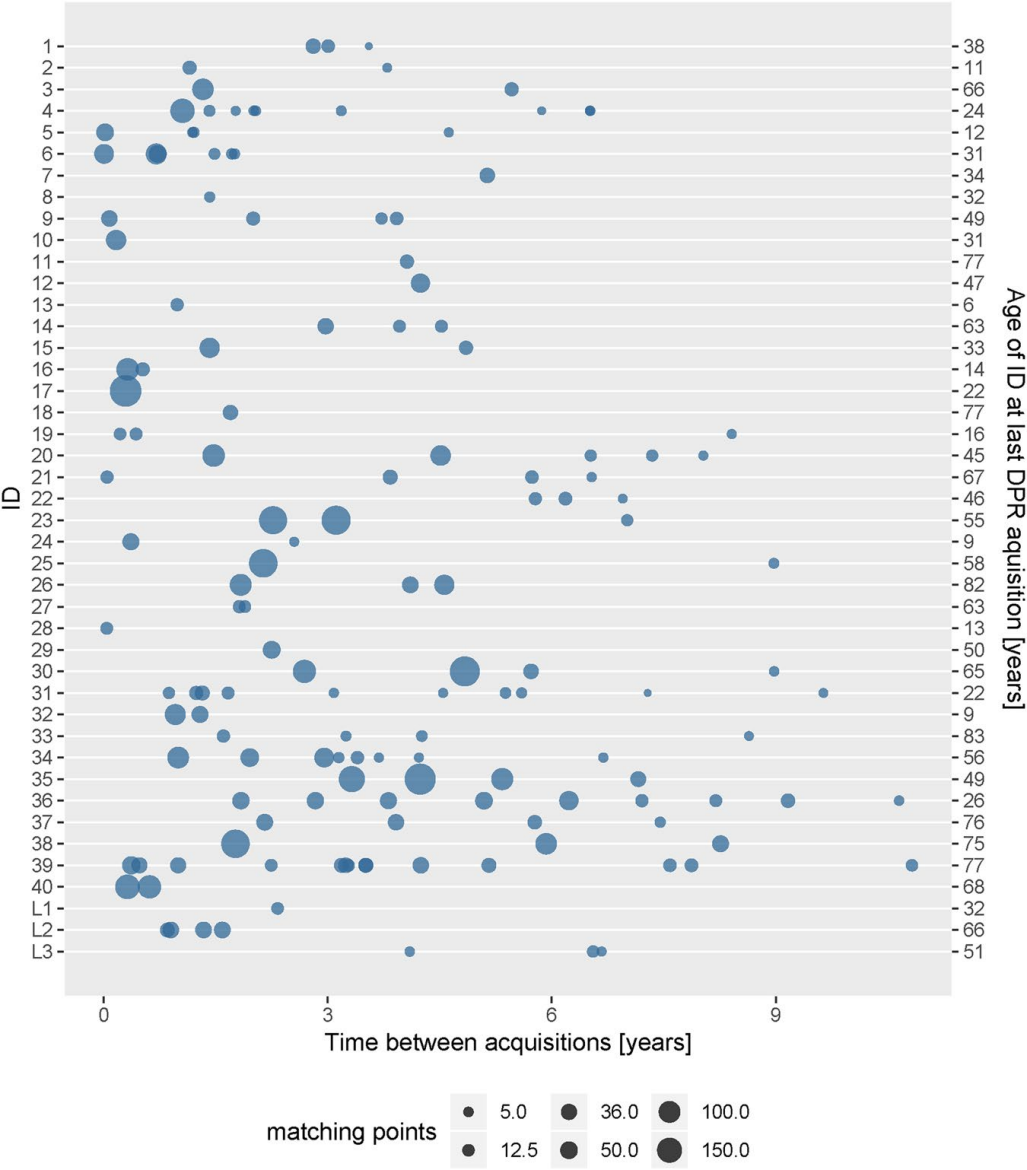
Overall results for DPR comparison shown for all 40 randomly selected persons and three corpses (L1–3) with filter setting E (compare with Table 1), with the exception of ID 28, L1 and L3, where the results of measurement series D, G and F are shown. Also shown is the relation of time between a DPR and the reference DPR of the same person. The size of the blob represents the number of matching points.

**Figure 3:**
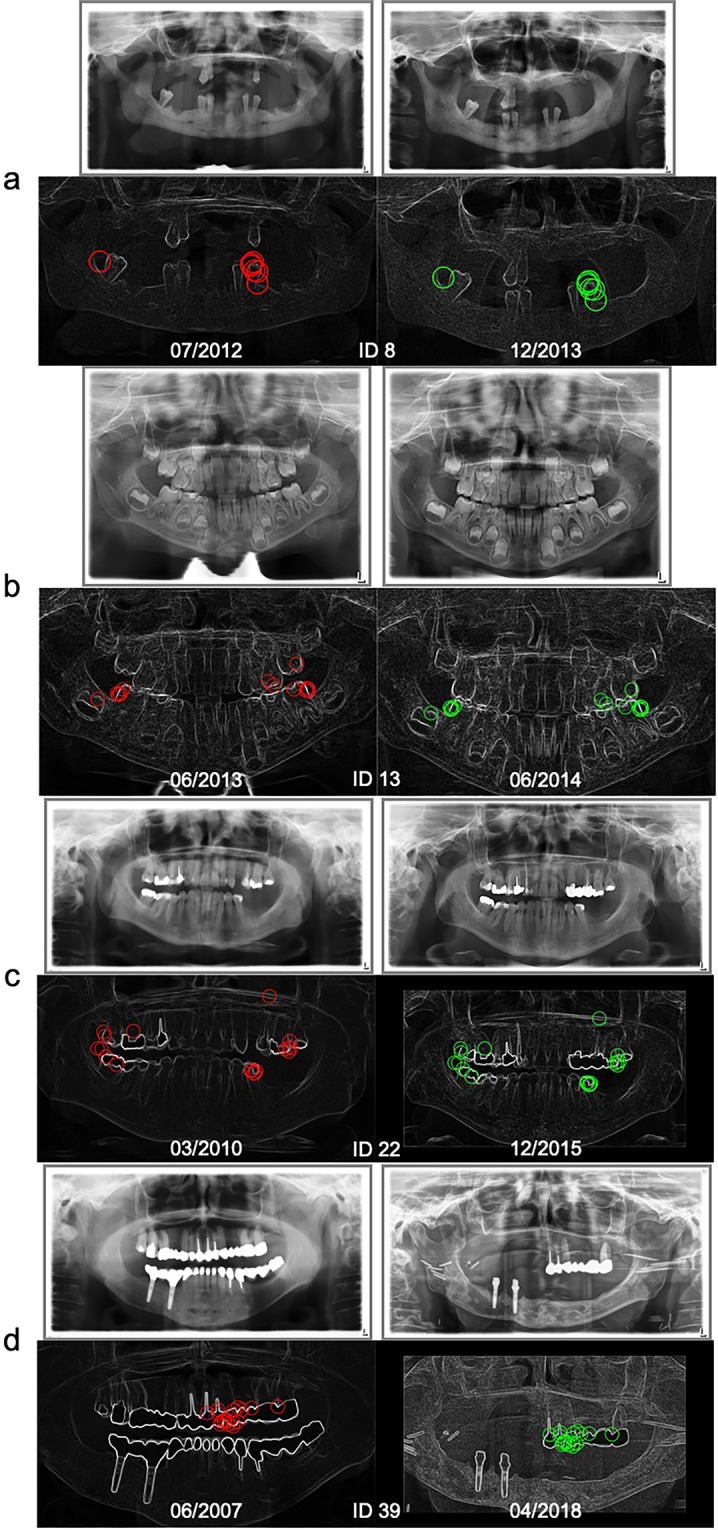
DPR examples of four test persons with and without dental works (**a-d**) and the related matching points for successful person identification – graphics with timestamp in the left column with matching points marked red and their identified counterparts with matching points marked green in the right column.

Fig. 2 shows the results for DPR-comparisons for each of the 40 randomly selected individuals related to time between acquisitions and the number of matching points (compare with Fig. 1). The test person ID 8 had only a few teeth without fillings or implants (see Fig. 3a). By contrast, the test person ID 22 had many fillings (see Fig. 3c). Successful identification was possible even if the time between acquisitions of two DPRs was nearly 11 years and when parts of the dentures were removed (see Fig. 3d, compare with Fig. 2 ID 39), or if only tooth edges were available, e.g., from a child (see Fig. 3b, compare with Fig. 2 ID 13).

In total, 111 of 138 images (80%) could be definitely identified with the filter settings used (see Table 1 and Table 3). The matching process can be complicated, resulting in few matching points for the same person where large changes of dentition appear, or in case of inferior image quality (low contrast) or non-standardized DPR acquisition (e.g. closed mouth). Furthermore, the detection rate for younger test persons can be lower compared to older individuals, because of the tooth growth. On the other hand, a large number of matching points can result if the search and reference images are very similar and, for example, dental works or implants are present.

**Figure 4:**
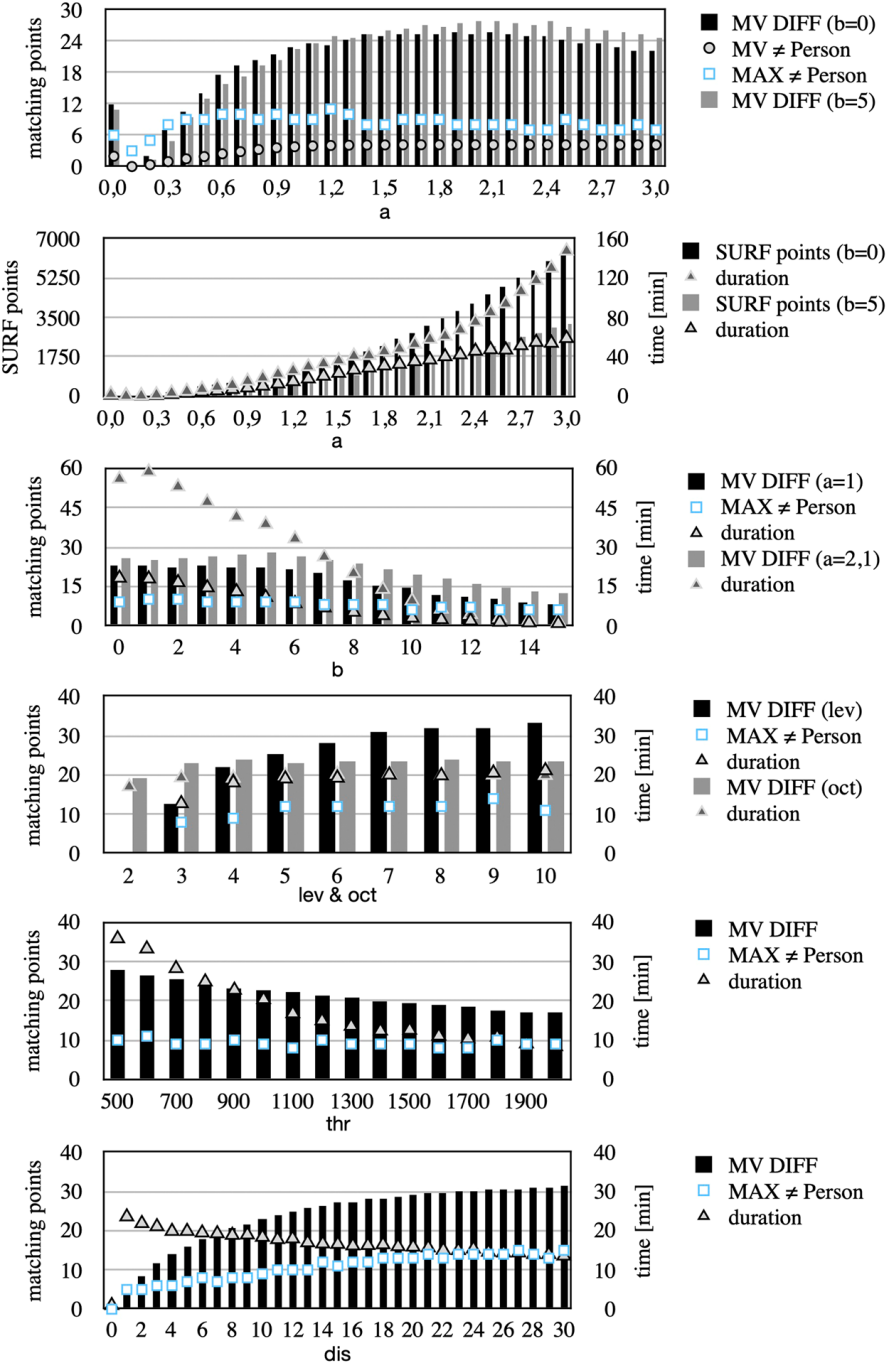
The result of systematic variations of the filter parameters (see Table 1) with MV DIFF = mean value difference of matching points for the same (=) identity and different (≠) identities, MAX = maximum matching points, SURF points and duration. The duration (signal processing time) is the time for 40 persons * 177 DPRs = 7080 comparisons.

Data processing (see Table 3) took between 8.56 ± 4.10 (filter setting A) and 298.94 ± 57.59 (filter setting E) minutes per person. The signal processing time depends directly on the database size and filter setting (see Fig. 4, compare Table 1 and Table 3). For all 10 test persons without reference in the database, the results were comparable. The maximum number of matching points is shown in Table 2.

Filter setting has a significant influence on the result. The systematic variations of the parameters (see Fig. 4 and Table 1) show that a larger Sobel filter size (a) allowed the number of SURF points to be increased. However, the signal processing time depends directly on the number of SURF points (features). By contrast, the noise filter size (b) reduced the number of SURF points. Ideally, it can be used to increase the number of matching points by filtering of noise, while signal processing time is reduced. A higher number of scale levels per octave (lev) can detect more features at finer scale increments, which results in more matching points for the same individual. In contrast, the number of octaves (oct) has low influence on the number of matching points found. A larger oct can detect larger features, which can be useful in some cases. A low value of metric threshold (thr) can improve the result; at the same time the maximum of matching points for non-identical individuals (threshold for unambiguous identification) is hardly changed. However, it has a large influence on the signal processing time. The maximum distance (dis) allowed between a point and the projection of its corresponding point, filter random sample consensus (RANSAC), has a particularly large influence on the maximum of matching points for different identities. The result can be better without considering the main orientation (rot) of the features. However, similar images of non-identical individuals also have more matching points, which can increase the maximum of matching points for different identities (e.g. see Table 2, filter setting E and E2). The signal processing time of the matching process can be reduced if the variable mat is set to 1 or 2 (see Tables 1 and 3). However, the consequence could be a slightly higher maximum of matching points for non-identical individuals.

With an optimal filter setting, the search and reference images can match better, which can make it easier for the SURF algorithm to find matching points. For example, by changing the parameter a, the edges are emphasized differently and the number of match points can change abruptly for the same person (see Fig. 1 right, middle). This also applies to comparisons between different persons, although the number of matching points is significantly lower (see MAX ≠ Person in Fig. 4 above).

### Post-mortem DPR acquisitions

**Figure 5:**
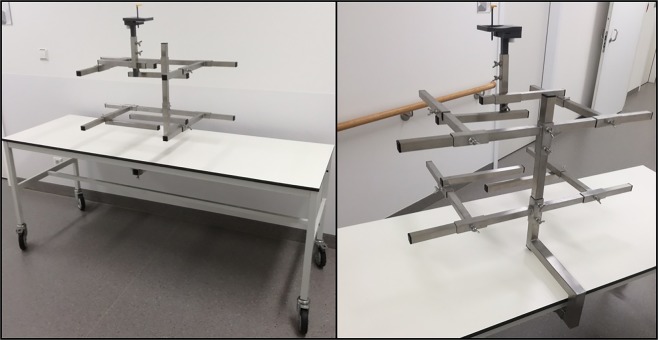
A fixation system developed for post-mortem DPR acquisitions. The upright and the holding arms can be adjusted so that both tall and short bodies can be fixed reliably. A chin rest helps to correctly position the head.

The fixation system permitted reliable fixation of corpses of every size (Fig. 5). Wing nuts enabled quick and easy adjustment of the holding arms to the body’s anatomy. The upright rod connected to the table was provided in two sizes so that, in case of short bodies, the X-ray unit would not collide with a high upright during rotation about the head.

**Figure 6:**
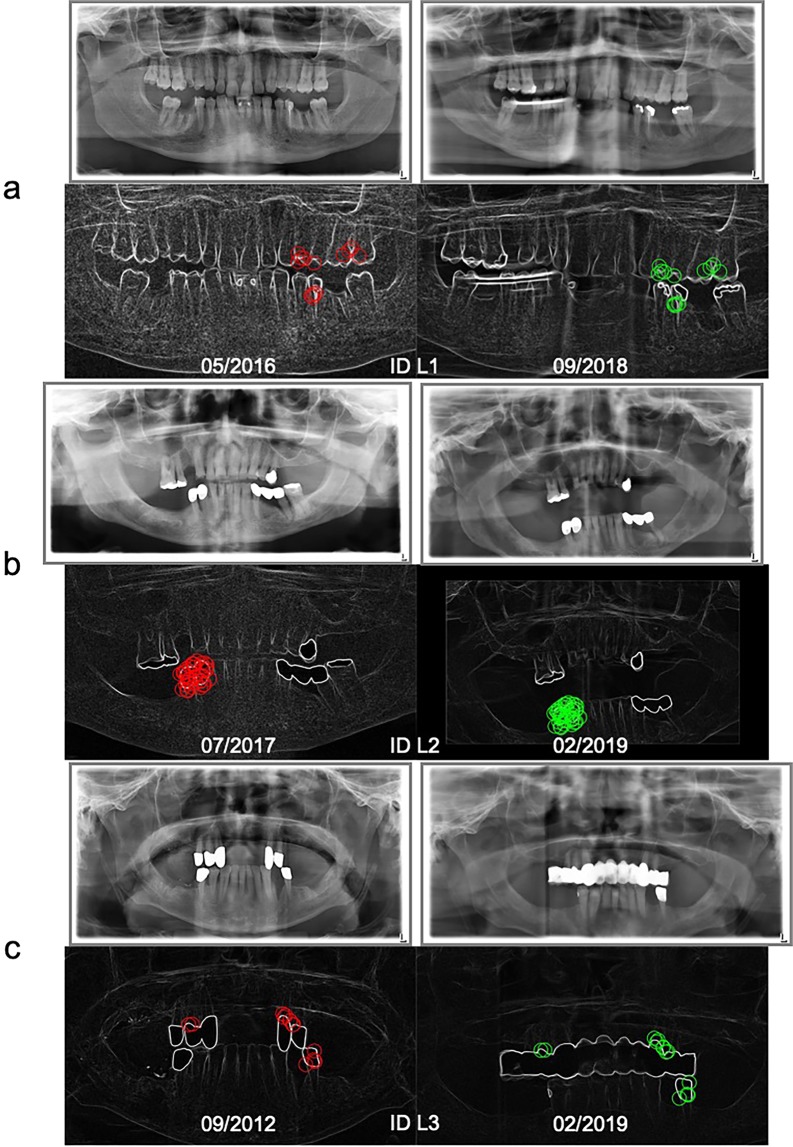
DPR examples of three corpses with dental works (**a**–**c**) and the related matching points for successful person identification: Graphics with timestamp in the left column with matching points marked red, and their identified counterparts with matching points marked green in the right column.

Acquiring post-mortem DPRs was successful in all cases (Fig. 6). During the recording of ID L1, a minor accidental movement of the head caused a signal distortion in the image (Fig. 6a right), because the corpse was measured with a first version of the fixation system without a chin rest. Nevertheless, a unique identification was possible in all cases (see Fig. 1 L1-L3 and Table 3). It was necessary, though, to optimize the filter for ID L1 (filter setting G or E2) and L3 (filter setting F or E2) to get an unambiguous result.

## Discussion

The experimental setup was designed to identify an unknown person, based on their DPR and using an automatic algorithm system. The tool proposed is able to filter large databases with many entries of probably matching partners (big data). Operating with an automatic DPR system and computer vision could be a useful and reliable tool for identification purposes. This identification method is suitable even if dental characteristics were removed or added in the past. Compared to the preliminary study^25^, the identification system has been further improved by an extension of the filter method and more precise cropping of the DPR.

A parameter for unambiguous identification (maximum number of matching points for non-identical individuals) has been found. This threshold is very important for the actual application of the automatic identification system. The threshold helps avoid a false-positive result if the searched person does not exist in the feature database (see Fig. 1, blue line). Filter setting E enables very robust identification. However, individual filter settings may be required for some cases (see Table 1 and Table 3). If, in case of more than one positive result, the threshold is not reached, the small group of potentially identical persons can be checked manually to increase identification certainty. Generally, qualified personnel for an individual assessment of the identity remains essential in forensic odontology.

In detail, the key factor to obtain those results is the combination of robust algorithms. The Sobel-Feldman operator^26^ as an edge detection algorithm calculates a rather inaccurate approximation of the image gradient, but the result is still of sufficient quality for person identification by DPR. It is a neighborhood-based gradient operator and performs a 2-D spatial gradient measurement on an image. The Sobel-Feldman operator can be extended to include all eight compass directions (rotated anti-clockwise by 45° increments). Furthermore, a modified Sobel-Feldman operator *G*_*a*_ was used to extract more potential features from the image. Thereby, a greater number of SURF points results in more matching points for images of the same person. Ideally, for non-identical individuals, a greater number of SURF points does not necessarily result in more matchings points. On the other hand, an edge filter with larger gradients results in more noise on the image, and furthermore, the greater number of SURF points to be compared in the matching process will increase the signal processing time. For this reason, an averaging filter can reduce noise as well as the number of non-essential SURF points. An averaging filter^27^ replaces each pixel value in an image with the mean value of its neighbors, including itself. This allows the elimination of pixel values that are unrepresentative of their surroundings.

The SURF algorithm from Herbert Bay^28–32^ allows fast and robust recognition of dental characteristics like tooth shapes, dental works (fillings, inlays, onlays, crowns, dental bridges, pivot teeth). The descriptor provides a unique and robust description of an image feature. Furthermore, the descriptor is invariant against scaling, rotation, illumination change, image noise, and, to a certain extent, perspective distortion^33,34^. The matching process performs a forward-backward match to select a unique match. Nevertheless, a swapping of the order of feature data sets (A = unknown person and B = database entry) can affect the result. Therefore, the matching process was performed twice with the order AB and BA, respectively.

The RANSAC algorithm^35^ is a valuable iterative method to estimate parameters of a mathematical model from a set of observed data that contains outliers. The matching process can be successful without using the RANSAC algorithm, but false-positive results will be conceivable, because there is no suitable threshold level related to the number of required matching points.

The acquisition of a post-mortem DPR requires technical and methodological prerequisites, which often are not given in forensic medicine. Therefore, a cooperation between forensic medicine and radiology is recommended. The post-mortem DPR needs to be comparable with an ante-mortem DPR. The acquisition of the post-mortem DPR is complicated due to handling of a corpse regarding the correct position, and rotation of the head. Additionally, the mouth must stay just slightly open during image acquisition. Moreover, due to the X-ray emission, the corpse cannot be held by an assistant but must be fixed in an upright position by suitable means while the X-ray tube and the detector rotate around the head of the corpse. For this reason we developed a fixation system that made it possible to acquire reliable post-mortem DPR records.

Computer vision is a powerful tool whose potential has not yet been fully recognized. For example, the number of SURF points and, thus, of the matching points found depends on pre-image-processing algorithms, whereby the result of computer vision can be significantly improved. Edge and noise filtering allows better tooth shapes recognition and enables successful identification also if there are just a few teeth or no characteristics (dental fillings, implants). The potential of the method presented here becomes clear especially in the case of ID L3 (Fig. 6c, top). At first sight, even a human eye cannot recognize any similarity. All the same, after optimization of the filter, the person was unmistakably identified (Fig. 1, right, L3). The matching points found make it plain that the subject indeed was the person searched for (Fig. 6c, bottom). Nevertheless, the matching process is based on small image details. Very large changes of teeth or dental works can complicate identification, e.g., where the shapes of the teeth have changed due to artificial prostheses, tooth growth or tooth extraction^9,36–39^. Furthermore, the unsuccessful identifications can possibly result from inferior image quality^3,6^. Dental characteristics could be insufficiently extractable in an overexposed radiograph^11^.

In summary, this study used a multiple algorithm software tool for person identification based on DPRs and yielded robust identification results for individuals, even for cases where dental characteristics changed over time. The quick identification through a large data set of DPRs creates a foundation for further research and development. With growing experience it will become clear which filter setting is the best for individual DPRs. Additionally, in connection with this study it turned out that the fast identification of unknown murder victims made it possible to convict the culprit.

## Methods

The study includes 61,545 DPRs of 33,206 persons, acquired between October 2006 and June 2018. The data sets (storage space 231 GiB) with the RIS service description “panoramic radiograph” were exported as Digital Imaging and Communications in Medicine (DICOM) from the local hospital picture archiving and communication system (PACS). All DPRs were filtered and evaluated on a standard pc (Intel® i7 3.1 GHz Quad-Core, 16 GB LPDDR3 2133 MHz) with Matlab R2018b (MathWorks^®^, Natick, Massachusetts, USA) including the toolboxes of image processing and computer vision system. All methods are approved by the local ethics committee of the Jena University Hospital (registration number 2019-1505-MV) and performed in accordance with its relevant guidelines and regulations. Written informed consent was waived by this committee, as it was a retrospective analysis of our usual everyday work.

### Image processing and feature database

For dental characteristics extraction, the following image processing steps were performed for all DPRs. The color depth was set to 8 bit (256 colors) to standardize all images. The image borders were cropped by 10 millimeter (mm) to remove the overexposed margins. The conversion between pixels and mm was calculated with the pixel spacing information from the DICOM header. Additionally, the top, left and right sides of the image were cropped to a final size of 180 ×100 mm (width x height) if the image size was large enough. A DPR can have different pixel densities (pixels per millimeter). With this procedure, all DPRs represent the same anatomical area independent of the image resolution.

Afterwards, a 3×3 Sobel filter^26^ was used for eight directional masks (0–315° step size 45°). The Sobel-Feldman operator *G* for an orientation of 0° is:

1 $${G}_{{0}^{^\circ }}=(\begin{array}{ccc}-1 & 0 & 1\\ -2 & 0 & 2\\ -1 & 0 & 1\end{array})$$

The direction of maximum contrast from black to white runs from left to right in the image. Furthermore, the Sobel filter was multiplied by the parameter *a* to get a modified Sobel-Feldman operator ${G}_{a,{0}^{^\circ }}$

2 $${G}_{a,{0}^{^\circ }}=(\begin{array}{ccc}-a & 0 & a\\ -2a & 0 & 2a\\ -a & 0 & a\end{array})$$

**Figure 7:**
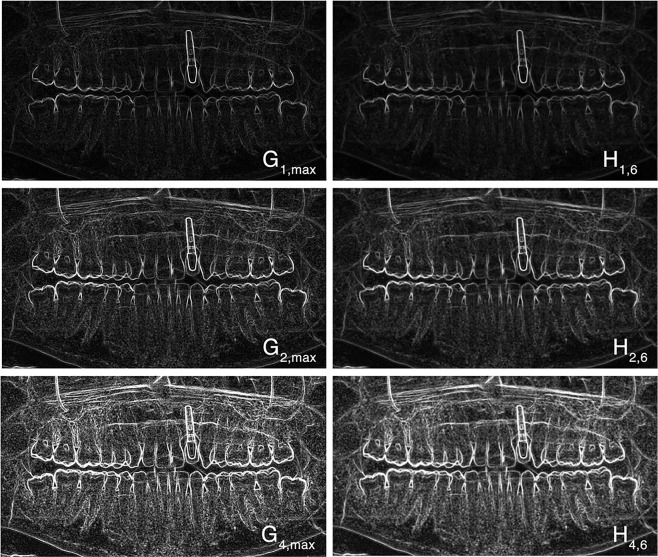
Maxima representation of an DPR after application of eight different Sobel operators for a value of *a* = 1, *a* = 2 and *a* = 4 (left) and additional application of an averaging filter with a size of *b* = 6 (right).

**Figure 8:**
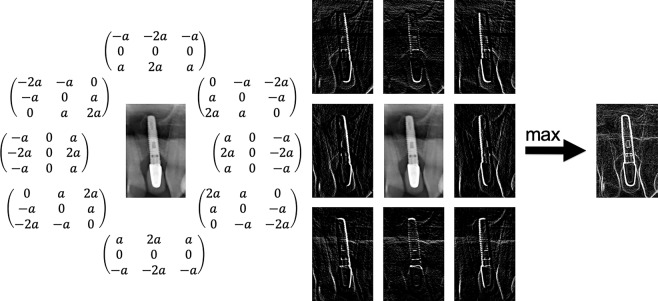
Shown on the left are eight modified Sobel-Feldman operators. The largest intensities of all eight direction images with *a* = 2 were used to create an image with emphasized edges (right).

The gradient of *G*_*a*_ can be changed with the parameter *a* (Fig. 7). The largest intensities of all eight direction images were used to create an image with emphasized edges (Fig. 8).

Furthermore, an averaging filter^27^ was used to reduce image noise. The function filters data by taking the 2-D convolution of the image and emphasizing edges, with the coefficient matrix *H* rotated 180 degrees. For example, a coefficient matrix with a size of three *H*_3_ is:

3 $${H}_{b=3}=(\begin{array}{ccc}0.1111 & 0.1111 & 0.1111\\ 0.1111 & 0.1111 & 0.1111\\ 0.1111 & 0.1111 & 0.1111\end{array})$$

Finally, the SURF algorithm^28–32^ was applied to find blob features. The SURF algorithm is based on the determinant of the Hessian matrix *M* with the convolution of the second order Gaussian derivative $L(x,y,\sigma )$ in the x, y and xy-directions (Laplacian of Gaussian) to identify features. To enhance performance, we calculated an approximation of the Laplacian of Gaussian $D(x,y,\sigma )$ using different box filters, which applied to the integral image of the DPR^28–32^:

4 $$Det(M)\approx \frac{{D}_{xx}(x,y,\sigma ){D}_{yy}(x,y,\sigma )-{(\beta {D}_{xy}(x,y,\sigma ))}^{2}}{{\sigma }^{2}}$$

*β* is the error correction for a filter box compared to a Gaussian derivative mask:

5 $$\beta =\frac{{\Vert {L}_{xy}(x,y,\sigma )\Vert }_{F}{\Vert {D}_{yy}(x,y,\sigma )\Vert }_{F}}{{\Vert {L}_{yy}(x,y,\sigma )\Vert }_{F}{\Vert {D}_{xy}(x,y,\sigma )\Vert }_{F}}\approx 0.9$$

where in ||*A*||_*F*_ is the Frobenius norm. A scale space is created by using box filters at different sizes (1^st^ octave 9×9, 15×15, 21×21, …, pixels; 2^nd^ octave 15×15, 27×27, 39×39, …, pixels; 3^rd^ octave 27×27, 51×51, 75×75, …, pixels; and so on). SURF introduces the notion of octaves, where each octave linearly samples only a small area of the scale space. The filtering and localization of scaling and rotation-invariant features in the image are done by a non-maximum suppression method; afterwards the determinant of the Hesse matrix is calculated as a Taylor extension^40^ up to the quadratic terms at the location of the feature. A feature is described by their coordinates, a main orientation, and a descriptor. The main orientation and the descriptor were determined by Haar wavelets to find gradients in the x and y directions. The SURF descriptor describes the pixel intensities within a scale-dependent neighborhood of each feature. The length of the descriptor was 64 for each feature. The pixels represent and match features specified by a single-point location^28,29^.

A dental characteristic database (feature database) was created with the free local server environment MAMP (version 5.3, appsolute GmbH, Germany) and MySQL (version 5.7.25, Oracle Corporation, USA). A MySQL table with 16 columns was created to save a unique person ID, image ID, the extracted DICOM header information (institution name, study date, patient ID, patient birth date, patient sex, accession number, study description) and the feature vectors and their corresponding locations. For that, the binary features object and the object for storing SURF interest points (point locations, scale at which the feature is detected, strength and orientation of the detected feature, sign of the Laplacian determined during the detection process, number of points held by the object) were saved in seven MySQL columns (longblob and one integer type).

### Identification process

The matching process was based on the SURF features to find unique corresponding points of interest between two DPRs (unknown person and database entry) that were rotated and scaled with respect to each other. For this, for each SURF point with the descriptor u (DPR of unknown person), two SURF points with the descriptors v_1_ and v_2_ (DPR of database entry) were searched that had the smallest Euclidean squared distance $d(u,v)$ to u:

6 $$d(u,v)=\mathop{\sum }\limits_{i=1}^{n}{({u}_{i}-{v}_{i})}^{2}$$

In the next step, weak matches with d > 0.4 were removed. A matching point was found wherever the nearest neighbor ratio:

7 $$\frac{{d}_{1}(u,{v}_{1})}{{d}_{2}(u,{v}_{2})}\le 0.6$$

was fulfilled. The function performs a forward-backward match to keep the best result. Finally, the result consists of indices, with each index pair corresponding to a match between a SURF point of the unknown person and one database entry (matching point). The number of matching points is an indicator for identification^28,29,34^.

Afterwards, the RANSAC algorithm^35^ was used to exclude outliers. RANSAC is an iterative method to estimate parameters of a mathematical model from a set of observed data that contains outliers. This allows the definition of a minimum necessary number of matching points (reliable identification threshold) and a maximum distance allowed between a point and the projection of its corresponding point. The corresponding projection is based on the estimated transform. The matching procedure was repeated in reversed order of binary feature objects (unknown person and database entry) - compare with variable *mat* in Table 1.

### Evaluation

For evaluation, 40 persons were randomly selected from the database. The selection criteria were based on the following requirements: The selected person had at least one other reference DPR in the database, and eight persons per age category (age younger than 18 yrs, 18 ≤ 33 yrs, 34 ≤ 53 yrs, 54 ≤ 67 yrs and older than 67 yrs) were selected. For each person, the most recent DPR acquisition was used for the matching process with the feature database. One selected DPR was compared with the remaining DPRs in the database. Eight different filter settings were used for matching with the content of the feature database (see Table 1). In addition, 10 individual data sets without another reference DPR were randomly selected (two per age category) to examine the possibility of false-positive results. The signal processing time for each person identification was measured. The influence of the filter parameter (see Table 1) was systematically investigated with all 178 DPRs of the 40 persons.

### Post-mortem DPR acquisitions

A fixation system was developed to support the post-mortem DPR acquisition with a Sirona Orthophos XG 3D (Dentsply Sirona Deutschland GmbH, Germany). As a table or base structure, a mobile bedstead was provided with a sterilizable wooden plate. A metal rail welded to the bottom side receives a slide-on fixation structure of appropriate height (depending on the body’s size). The structure consists of a vertical rod to which a total of ten horizontal arms can be flexibly attached to fix the body on the table in a seated position. A chin rest, which can be adjusted vertically and laterally, supports the head. Also provided is a teeth rest permitting separation of the upper and lower teeth. By means of this fixation system we acquired three post-mortem DPRs.
